# Transcriptome analysis of fat accumulation in 3T3-L1 adipocytes induced by chlorantraniliprole

**DOI:** 10.3389/fnut.2022.1091477

**Published:** 2022-12-15

**Authors:** Ge Chen, Ge Wang, Weidong Xu, Ying Xiao, Ye Peng

**Affiliations:** ^1^School of Food and Biological Engineering, Jiangsu University, Zhenjiang, Jiangsu, China; ^2^School of Pharmacy, Jiangsu University, Zhenjiang, Jiangsu, China; ^3^Faculty of Medicine, Macau University of Science and Technology, Taipa, Macao SAR, China

**Keywords:** chlorantraniliprole, adipogenesis, fat accumulation, RNA sequencing, transcriptome analysis

## Abstract

**Introduction:**

Chlorantraniliprole is a diamide insecticide widely used in agriculture. Chlorantraniliprole has been previously found to increase the accumulation of triglycerides (fats) in adipocytes, however, the underlying molecular mechanism is unknown. The present study aimed to explore the molecular mechanisms of chlorantraniliprole-induced fat accumulation in 3T3-L1 adipocytes.

**Methods:**

We measured the triglyceride content in chlorantraniliprole-treated 3T3-L1 adipocytes, and collected cell samples treated with chlorantraniliprole for 24 h and without any treatment for RNA sequencing.

**Results:**

Compared with the control group, the content of triglyceride in the treatment group of chlorantraniliprole was significantly increased. The results of RNA sequencing (RNA-seq) showed that 284 differentially expressed genes (DEGs) were identified after treatment with chlorantraniliprole, involving 39 functional groups of gene ontology (GO) and 213 KEGG pathways. Moreover, these DEGs were significantly enriched in several key genes that regulate adipocyte differentiation and lipogenesis including *Igf1, Rarres2, Nr1h3*, and *Psmb8*.

**Discussion:**

In general, these results suggest that chlorantraniliprole-induced lipogenesis is attributed to a whole-gene transcriptome response, which promotes further understanding of the potential mechanism of chlorantraniliprole-induced adipogenesis.

## 1 Introduction

In modern agriculture, insecticides have been widely used, and the use of pesticides gradually from organophosphorus, pyrethroid, and carbamate to nicotine and diamide pesticides (1, 2). Chlorantraniliprole (a diamide insecticide) acts on the ryanodine receptor (3). As an excellent ovicidal and larvicidal agent, chlorantraniliprole has a good control effect against lepidopteran pests and is used to control a variety of pests on fruits, vegetables, and grains (4–8).

More and more studies are reporting the potential link between pesticides exposure and obesity (9–18). Our previous study has shown that chlorantraniliprole may enhance adipogenesis in 3T3-L1 adipocytes through inhibiting AMPK (19). At present, however, the mechanism of action and regulatory system of chlorantraniliprole on fat accumulation are not yet clear.

Ribonucleic acid (RNA) sequencing is a powerful method in understanding the differential gene expression and potential significantly enriched pathways, which are critical in understanding the underlying molecular mechanisms (20, 21). Therefore, we investigated the potential molecular mechanism of lipogenesis in chlorantraniliprole-treated 3T3-L1 adipocytes by transcriptomics.

## 2 Materials and methods

### 2.1 Materials and reagents

Murine 3T3-L1 cells were provided by Shanghai Cell Bank of the Chinese Academy of Sciences (Shanghai, China). Dulbecco modified Eagle medium (DMEM), Calf serum (CS), fetal calf serum (FBS), insulin, dimethyl sulfoxide (DMSO), dexamethasone and isobutyl methyl xanthine (IBMX) were all from Sigma-Aldrich Company (St. Louis, Missouri, USA). RevertAid First Strand cDNA Synthesis Kits were provided by Thermo Fisher Scientific (Rockford, IL). SYBR^®^ Green Master Mix was provided by TIANGEN BIOTECH (Beijing, China). Chlorantraniliprole is provided by J&K Scientific Ltd. (Shanghai, China). Phosphatase inhibitor and phosphate buffered saline (PBS) were provided by Beyotime Biotechnology Co., Ltd. (Shanghai, China). TRIzol reagent was provided by Thermo Fisher Scientific Co., Ltd. (Shanghai, China).

### 2.2 3T3-L1 preadipocytes culture

The method for culture 3T3-L1 preadipocytes was determine based on previous studies (9, 16). 3T3-L1 preadipocytes were maintained at 37°C in a carbon dioxide incubator in DMEM containing 10% FBS. Two days after confluency (Day 0), 3T3-L1 preadipocytes differentiated in a mixture of isobutyl methyl xanthine (0.5 mM), dexamethasone (0.1 μM) and insulin (1 μg/mL) in DMEM containing 10% FBS. Two days later (Day 2), the medium was replaced with a DMEM solution containing insulin (1 μg/mL) in 10% FBS. Starting on Day 4, the cells were differentiated in DMEM containing 10% FBS and changed every 2 days until the end of differentiation. Starting on Day 0, cells were treated with 0.02% DMSO (as control) or 10 μM chlorantraniliprole in DMSO. The concentration of chlorantraniliprole used was determined by reference to previous studies (19).

### 2.3 Determination of triglyceride content

Chlorantraniliprole-treated cells after 8 days of differentiation sucked off the culture medium, washed twice with PBS, and then added PBS to scrape off the bottom cells. Based on the instructions provided by the manufacturer, use the kit of Nanjing Jiancheng Biotechnology Research Institute (Nanjing, Jiangsu, China) to determine the content of triglyceride (TG) and protein. The TG content was standardized with protein concentration.

### 2.4 RT-qPCR experiment of chlorantraniliprole with different treatment time

3T3-L1 adipocytes differentiated for 24, 48, and 96 h, and 8 days were completely digested with TRIzol reagent, collected, and then stored in the −80°C refrigerator after quick-frozen in liquid nitrogen. Based on the manufacturer’s instructions, total RNA was extracted without RNase and then reverse transcribed into cDNA using the RevertAid First Strand cDNA Synthesis Kit. The RT-qPCR consisting of SYBR Green Master Mix was then performed on a StepOne Plus real-time PCR system (Applied Biosystems, Carlsbad, CA). The primers related to lipogenesis (*FGF10, FAS, PPAR*γ, *SCD1* and *SNAI2*) were used to evaluate the effect of lipogenesis induction of adipocytes at different differentiation times. With GAPDH as internal reference gene, the expression level of target gene was standardized. The expression of target genes was quantitated with the 2^(–Δ^ ^Δ^ ^CT)^ calculation method.

### 2.5 Samples collection and library construction

The cells were divided into the control group and chlorantraniliprole treatment group. After differentiation and culture for 24 h, the cells were fully digested with TRIzol reagent, and immediately after collection, they were quickly frozen in liquid nitrogen and stored at –80°C. The collected samples were sent to Gene Denovo Biotechnology CO (Guangzhou, China) for RNA library construction.

### 2.6 Raw data processing of RNA-seq

In order to assure the quality of the data, the raw data were filtered before the information analysis. Filtering out the raw data containing adapter, the n ratio being more than 10% or all a bases and the low-quality reads (the number of bases with a mass value q being less than or equal to Q ≤ 20 accounts for more than 50% of the whole read) to obtain clean reads. The ribosomal-matched reads were removed without allowing mismatching and the retained unmapped reads were used for subsequent transcriptomics analysis.

### 2.7 Differentially expressed genes (DEGs) and biological function analysis

The key to understand and recognize phenotypic variation is to correctly identify differentially expressed genes (DEGs) under specific conditions (22). The reads count data obtained from gene expression level analysis was analyzed by DESeq2 software (23). Based on the screening condition *P* < 0.05 and fold change > 1.5, the significant DEGs between the corresponding samples could be obtained. DEGs was then subjected to Gene Ontology (GO) enrichment analysis and KEGG pathways analysis.

### 2.8 Statistical analyses

All results are expressed as means ± SEM. Data were analyzed using the GraphPad Prism version 9.2.0. Tukey’s multiple-range test was used to determine significant differences between treatments. For all statistical analyses, *P-*value < 0.05 were considered significant, *, *P* < 0.05; **, *P* < 0.01.

## 3 Results

### 3.1 Chlorantraniliprole increases fat accumulation in 3T3-L1 preadipocytes

**FIGURE 1:**
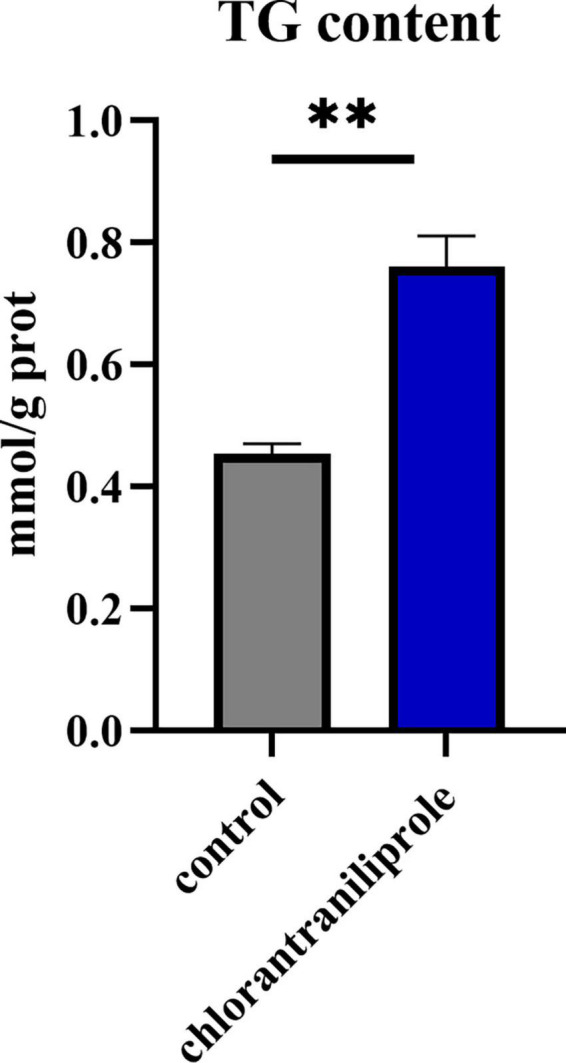
The triglyceride content in the C-vs.-CH is detected by a TG kit. C represents four untreated control samples; CH represents four chlorantraniliprole-treated treatment group samples. Numbers are mean ± SEM (*n* = 4). **P < 0.01.

As shown in Figure 1, compared with the control group, the content of triglyceride in the treatment group of 10 μM chlorantraniliprole was significantly increased (*P* < 0.01). This is consistent with our previous report (19) and suggests a significant effect of chlorantraniliprole on fat accumulation.

### 3.2 Effects of different treatment time of chlorantraniliprole on the expression of adipogenic regulatory factors

**FIGURE 2:**
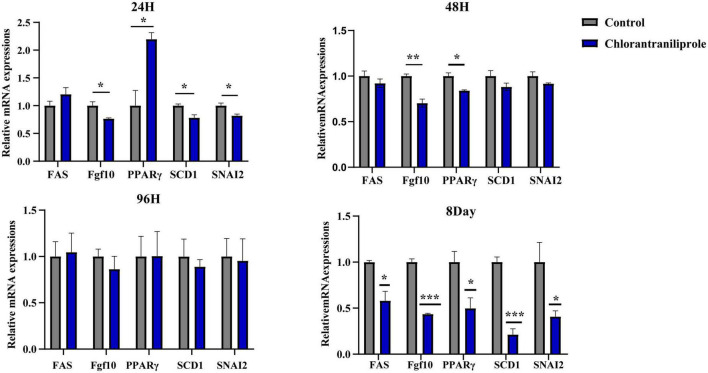
Relative mRNA expression of the selected genes by RT-qPCR. C represents three untreated control samples; CH represents three chlorantraniliprole-treated treatment group samples. Numbers are mean ± SEM (*n* = 3). *P < 0.05, **P < 0.01, ***P < 0.001.

As shown in Figure 2, compared with the control group, after 24 h treatment with chlorantraniliprole, the expression of fatty acid synthetase (*FAS*) was increased, the expression of peroxisome proliferator-activated receptor γ (*PPAR*γ) was significantly increased, and the expressions of fibroblast growth factor 10 (*FGF10*), stearyl coenzyme A dehydrogenase-1 (*SCD1*), and snail family transcriptional repressor 2 (*SNAI2*) were significantly decreased. After 96 h of treatment, there are no significant differences between the expression levels of these genes. However, after 48 h and 8 day of treatment, the expression levels of all genes were decreased, compared to 48 h of treatment, the expression of *FGF10* and *PPAR*γ decreased significantly, while on 8 days of treatment all gene expression levels were decreased significantly. Since chlorantraniliprole at 24 h significantly increased the expression of *PPAR*γ, a key regulator of adipogenesis. The following transcriptome analysis was performed with cells treated with chlorantraniliprole for 24 h.

### 3.3 Global changes of transcriptome in adipocytes induced by chlorantraniliprole

As shown in Table 1, after the low-quality reads and adapter were removed, more than 36823012 clean reads were generated per library. Compared with the ribosome database, the mapping reading rate of each library was less than 0.2%, and the total mapping gene rate of total genes compared with the reference genome was higher than 94.74%. In base quality analysis, the ratio of guanine to cytosine (GC content) of each bank and the ratio of raw data Q20 were 49.02–49.43 and 94.84–95.68%, respectively.

**TABLE 1:** Output statistics of sequencing reads and mapping results.

Sample	C1	C2	C3	CH1	CH2	CH3
Clean data	44,317,326	45,764,842	44,217,584	36,823,012	41,852,734	40,239,766
Adapter	11,016	11,868	11,986	9,214	11,292	10,348
Low quality	190,094	193,148	193,648	171,708	168,250	197,752
mRNA mapped reads (%)	0.18%	0.19%	0.20%	0.19%	0.19%	0.20%
Total mapped (%)	94.92%	95.56%	95.92%	94.85%	95.30%	94.74%
Raw data (bp)	6,677,765,400	6,895,478,700	6,663482,700	5,550,590,100	6,304,841,400	6,067,179,900
Before filter_Q20 (%)	94.86%	95.24%	95.50%	94.77%	95.05%	94.65%
Before filter_GC (%)	49.23%	49.15%	49.34%	49.37%	49.46%	49.06%
Clean data (bp)	6,635,565,727	6,851,231,394	6,617,495,837	5,513,660,389	6,65,540,360	6,025,633,944
After filter_Q20 (%)	95.03%	95.41%	95.68%	94.95%	95.22%	94.84%
After filter_GC (%)	49.20%	49.11%	49.31%	49.34%	49.43%	49.02%

C-1, C-2, and C-3 are three untreated control samples; CH-1, CH-2, and CH-3 are three chlorantraniliprole-treated treatment group samples.

### 3.4 DEGs analysis of transcriptome data

**FIGURE 3:**
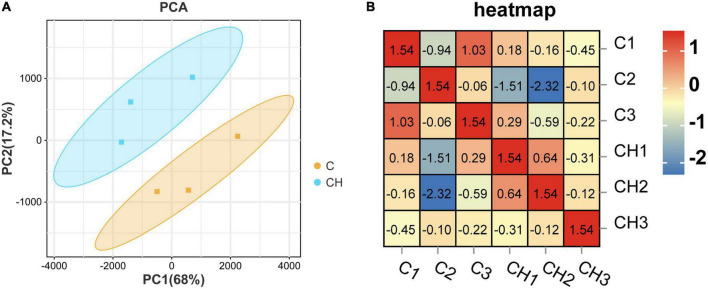
Principal component analysis (PCA) **(A)** and correlation heat map **(B)** of sample relationship in C-vs.-CH. C represents three untreated control samples; CH represents three chlorantraniliprole-treated treatment group samples.

**FIGURE 4:**
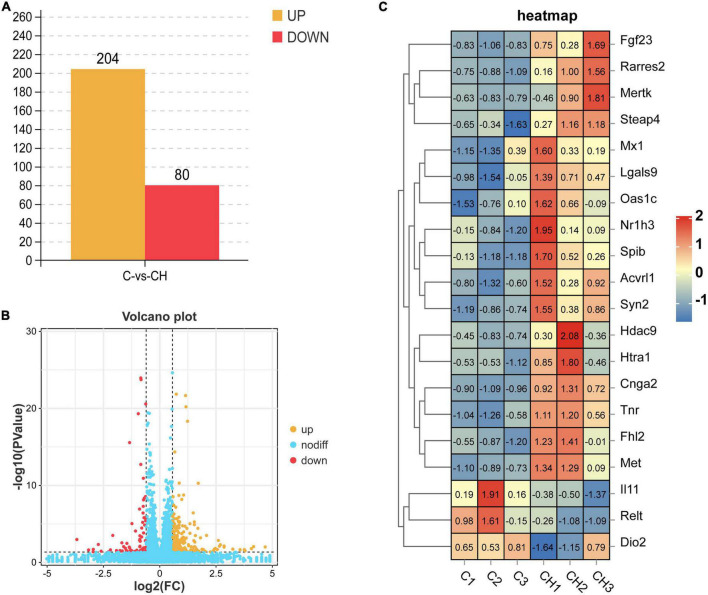
Holistic statistics **(A)**, volcano plot analysis **(B)**, and correlation heatmap **(C)** of DEGs in C-vs.-CH. C represents three untreated control samples; CH represents three chlorantraniliprole-treated treatment group samples.

Figure 3A shows the principal component analysis (PCA) of the samples with principal component 1 (PC1) at 68% and PC2 at 17.2%. Figure 3B is a heat map of that correlation coefficient of the sample. PCA analysis and correlation coefficient showed that chlorantraniliprole treatment induced clear transcription separation, and ensured that the data were used for further functional analysis. The results of DESeq2 showed that 284 genes were differentially expressed (*P* < 0.05 and fold change > 1.5), of which 204 genes were up-regulated and 80 genes were down-regulated (Figure 4A). The volcano gram shows the distribution of DEGs between the control (C) and chlorantraniliprole treated (CH) groups (Figure 4B). Up-regulated genes are shown in yellow, and down-regulated genes are shown in red. Hierarchical clustering was performed on the differential gene expression patterns, and the clustering results were presented using the heat map. Z-score was applied to each gene, and the first 20 up-regulated and down-regulated genes were subjected to hierarchical clustering and heat mapping (Figure 4C).

### 3.5 GO and KEGG analysis of DEGs

**FIGURE 5:**
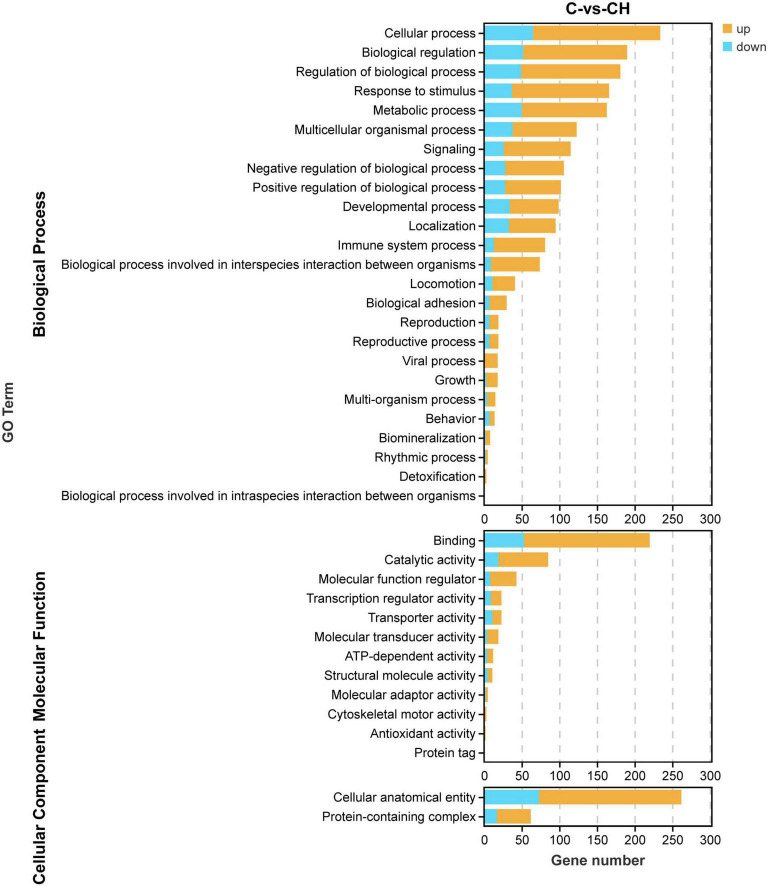
Gene ontology analysis of DEGs in C-vs.-CH. C represents three untreated control samples; CH represents three chlorantraniliprole-treated treatment group samples.

**FIGURE 6:**
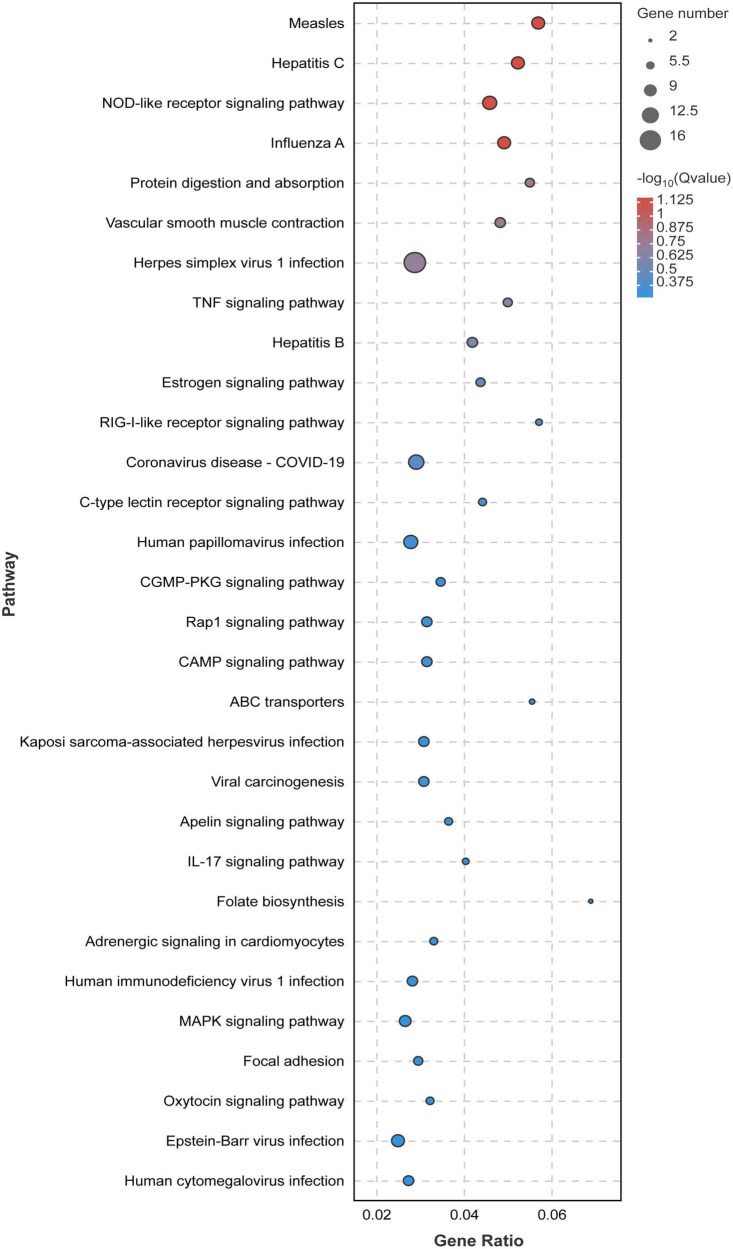
Significant bubble diagram for top thirty enrichment in the KEGG pathway. Rich factor is the ratio of the DEGs number to the background number in the certain pathway. The size of the dots represents the number of genes, and the dots represents the range of the *q*-value. C represents three untreated control samples; CH represents three chlorantraniliprole-treated treatment group samples.

In order to study the influence of chlorantraniliprole treatment on different biological functions of DEGs in adipocytes, DEGs was analyzed and identified through GO enrichment. As shown in Figure 5, GO enrichment divided 284 DEGs into 39 functional groups, with the vertical coordinate as the secondary GO term, and the horizontal coordinate as the number of differential genes in the term, yellow as up-regulated and blue as down-regulated. In the analysis of KEGG pathways, DEGs involved 213 pathways, and the analysis of the top 30 enriched KEGG pathways mainly included TNF signaling pathway, CAMP signaling pathway, IL-17 signaling pathway, and MAPK signaling pathway (Figure 6). GO enrichment and KEGG pathway analysis indicated that chlorantraniliprole treatment had an influence on different gene networks in adipocytes.

### 3.6 Candidate DEGs related to adipocyte differentiation and lipid metabolism

**FIGURE 7:**
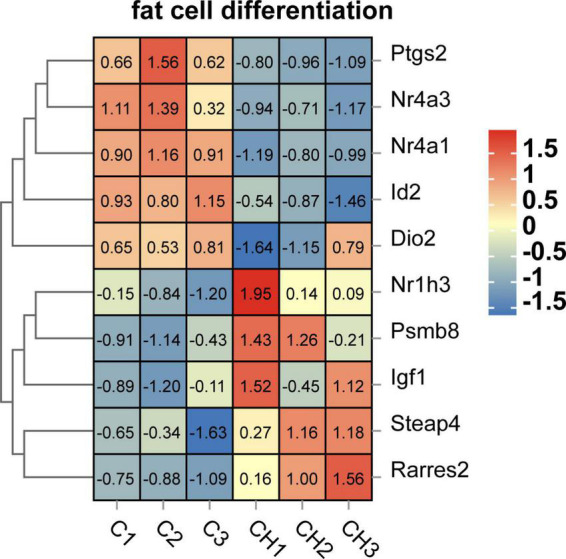
Heat map related to adipocyte differentiation function of DEGs in C-vs.-CH. C represents three untreated control samples; CH represents three chlorantraniliprole-treated treatment group samples.

Screened by biological process of GO enrichment, GO term related to lipid accumulation includes adipocyte differentiation, lipid metabolism and circadian rhythm. As shown in Figure 7, several key genes regulating adipocyte differentiation were significantly up-regulated by chlorantraniliprole. Above all things, insulin-like growth factor 1 (*IGF1*), retinoic acid receptor responder 2 (*RARRES2*), nuclear receptor subfamily 1 group H member 3 (*NR1H3*), and proteasome 20S subunit beta 8 (*PSMB8*) are highly expressed, and these genes are reported to be involved in regulating adipocyte differentiation and promoting fat accumulation (24–27).

## 4 Discussion

In this study, the results of chlorantraniliprole-induced fat accumulation were consistent with our previous studies (19). 284 DEGs were found through transcriptome analysis, including 204 up-regulated genes and 80 down-regulated. Besides, functional enrichment resulted in 39 functional groups and 213 KEGG pathways. Further, we identified four DEGS (*IGF1, RARRES2, NR1H3, PSMB8*) that correspond to adipocyte differentiation function (GO term).

*IGF1* is a key factor to regulate adipocyte differentiation and lipid accumulation. The differentiation and metabolic regulation of *IGF1* signaling adipocytes may be linked with the activation of downstream AMPK pathway by insulin receptor substrate (IRS) protein (25, 28). In addition, our previous research showed that chlorantraniliprole induced adipogenesis in adipocytes through AMPKα pathway (19). The current results indicate that *IGF1* may have an influence in in chlorantraniliprole-induced adipogenesis.

*RARRES2*, also known as Chemerin, is a new type of adipokine, which regulates adipogenesis and adipocyte metabolism by activating chemokine-like receptor 1 (CMKLR1) (24). It has been proved that down-regulation of chemerin damages 3T3-L1 adipogenesis and the biological function of adipocytes (29), chlorantraniliprole therefore may promote 3T3-L1 cell differentiation via promoting the expression of *RARRES2*.

*NR1H3*, also known as Liver X receptor alpha (LXRα). Studies have shown that *NRIH3* is up-regulated by *PPAR*γ agonists in mature 3T3-L1 adipocytes (26), which is consistent with our current results that chlorantraniliprole increase the expression of *PPAR*γ to upregulate the expression of LXRα in adipocytes.

*PSMB8* is a multi-catalytic protease complex, which regulates the differentiation of preadipocytes and is necessary for preadipocytes to differentiate into adipocytes (27). Previous studies have indicated that the down-regulation of *PSMB8* in 3T3-L1 adipocytes inhibits adipocyte differentiation, while other studies have shown that the immune proteasome activity mediated by *PSMB8* is the direct regulator of preadipocyte differentiation and its final maturation (27, 30). The current results are compatible with the above reports, that chlorantraniliprole may promote fat formation by increasing *PSMB8* expression in adipocytes.

Circadian rhythm is an important regulatory system maintaining the dynamic balance of normal cells and tissues, which is regulated by the biological clock (31). The destruction of biological clock can cause disorder of circadian rhythm and metabolic diseases such as type 2 diabetes and obesity (32). Previous studies have shown that circadian rhythm disorders destroy insulin sensitivity, leading to insulin resistance and obesity (32, 33). *ID2* (inhibitor of DNA binding 2), is an important transcriptional inhibitor that was reported to regulate expression in the circadian rhythm of mouse adipose tissues (34). Compared with wild-type mice, the *ID2* gene knockout mice showed corresponding physiological disorders in lipid regulation, and at the same time, the ID protein effectively inhibited the CLOCK-BMAL1 transactivation of the clock gene and the activity of the clock control gene (35). Furthermore, the possible mechanism of enhancing insulin sensitivity in *ID2*−/− mice was reported (36). These studies together with our current results that chlorantraniliprole reduces the expression of *ID2* suggested that chlorantraniliprole may reduce *ID2* to promote fat accumulation.

To sum up, the effects of chlorantraniliprole on lipogenesis in 3T3-L1 cells were investigated by transcriptomics of RNA-seq. Chlorantraniliprole may affect the pathways related to adipocyte differentiation and circadian rhythm to promote adipogenesis. At present, however, our study is limited to *in vitro* cell culture models, and further *in vivo*, epidemiological, and western blotting studies are necessary to further explore the association between chlorantraniliprole and obesity.

## Data availability statement

The original contributions presented in this study are publicly available. This data can be found here: https://www.ncbi.nlm.nih.gov/bioproject/PRJNA898515.

## Author contributions

GC, GW, and YP contributed to the conception or design of the works. GC and GW were responsible for the data acquisition, analysis, or interpretation. GC was responsible for the article writing. YP was responsible for the revision of the works. All authors participated in the reading of the manuscript and approved the submitted version.
